# Infrared Studies on Bimetallic Copper/Nickel Catalysts Supported on Zirconia and Ceria/Zirconia

**DOI:** 10.1007/s10562-013-1001-y

**Published:** 2013-04-06

**Authors:** Astrid Kitla, Olga V. Safonova, Karin Föttinger

**Affiliations:** 1 Institute of Materials Chemistry, Vienna University of Technology, Getreidemarkt 9 BC01, 1060 Vienna, Austria; 2 Paul Scherrer Institut, 5232 Villigen PSI, Switzerland

**Keywords:** CuNi alloy, Bimetallic catalyst, Methane conversion, Coke, Infrared spectroscopy, CO adsorption

## Abstract

**Abstract:**

Infrared spectroscopy has been employed for a detailed characterization of ZrO_2_ and CeO_2_/ZrO_2_ supported nickel and copper/nickel catalysts to be utilized for methane decomposition. Adsorption of CO at 303 K was performed in order to determine the surface composition and accessible adsorption sites. Alloy formation occurred during reduction, as indicated by a red-shift of the vibrational band of CO on Ni: by 27 cm^−1^ on nickel-rich CuNi alloy, by 34 cm^−1^ on 1:1 Cu:Ni and by 36 cm^−1^ on copper-rich CuNi alloy. CuNi alloy formation was confirmed by X-ray absorption spectroscopy during reduction revealing a considerably lower reduction temperature of NiO in the bimetallic catalyst compared to the monometallic one. However, hydrogen chemisorption indicated that after reduction at 673 K copper was enriched at the surface of the all bimetallic catalysts, in agreement with IR spectra of adsorbed CO. In situ IR studies of methane decomposition at 773 K demonstrated that the addition of Cu to Ni strongly reduced coking occurring preferentially on nickel, while maintaining methane activation. Modification of the zirconia by ceria did not have much effect on the adsorption and reaction properties. Ceria-zirconia and zirconia supported samples exhibited very similar properties and surface chemistry. The main difference was an additional IR band of CO adsorbed on metallic copper pointing to an interaction of part of the Cu with the ceria.

**Graphical Abstract:**

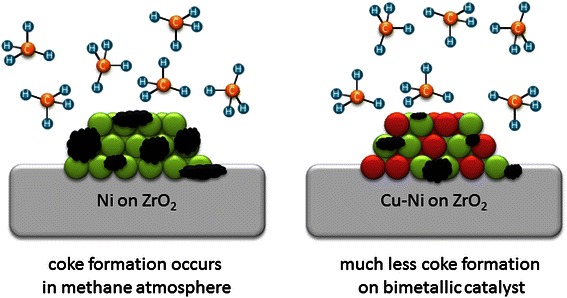

## Introduction

The global need of energy, rising oil prices and environmental requirements to reduce CO_2_ emissions increase the interest in alternative energy generation, such as fuel cells as clean and efficient means of energy production. There are a number of different types of fuel cells with those operating via a proton-exchange membrane receiving the largest attention [1]. Fuel cells that run directly on hydrogen are considered clean because the exhaust is only water, but this ignores the fact that the vast majority of H_2_ is generated by reforming of hydrocarbons, such as methane, [2] producing CO or CO_2_ by-product. Furthermore, the storage of gaseous hydrogen or liquefied hydrogen is difficult and dangerous. Thus, the production of hydrogen from hydrocarbons by internal reforming in solid oxide fuel cells (SOFCs) represents a good alternative.

Catalysis plays an important role during all involved processes. Catalysts are used for the production of H_2_ from hydrocarbons (preferentially from sustainable sources), and catalysts are also used directly in solid oxide fuel cells for e.g. methane reforming or oxygen activation. Methane as a feedstock can be obtained from different sources, such as from natural gas or biogas. H_2_ can then be produced by methane steam reforming [3–5], (1), dry reforming [4, 6–13], (2) or partial oxidation [14, 15], (3) of methane.

1 $$ {\text{CH}}_{ 4} \, + {\text{ H}}_{ 2} {\text{O }} \rightleftarrows {\text{CO }} + {\text{ 3H}}_{ 2} \quad \Updelta H^\circ_{{ 2 9 8 {\text{K}}}} = - 20 6 {\text{kJ}}/{\text{mol}} $$

2 $$ {\text{CH}}_{ 4} \, + {\text{ CO}}_{ 2} \rightleftarrows {\text{ 2CO }} + {\text{ 2H}}_{ 2} \quad \Updelta H^\circ_{{ 2 9 8 {\text{K}}}} = + 2 6 1 {\text{kJ}}/{\text{mol}} $$

3 $$ {\text{CH}}_{ 4} \, + \, \frac{1}{2}{\text{O}}_{ 2} \, \to {\text{CO }} + {\text{ 2H}}_{ 2} \quad \Updelta H^\circ_{{ 2 9 8 {\text{K}}}} = - 3 6 {\text{kJ}}/{\text{mol}} $$

Commercially used catalysts for methane reforming are based on Ni. The active nickel particles, however, tend to form coke, leading to deactivation of the catalysts [16]. Improved stability against coke formation was observed for NiAu catalysts [17–19] and was explained by the reduced stability of adsorbed carbon on nickel atoms in the vicinity of a gold atom. In the current work Cu was used to dilute Ni, in order to improve the stability against coke formation while maintaining the high activity of nickel catalysts for methane activation. The interaction of Ni with methane is the key step in methane reactions and of fundamental importance in coke formation. Methane decomposition was therefore examined in the absence of H_2_O, CO_2_ or O_2_.

Liao et al. [20] calculated dissociation enthalpies and activation energies of the sequential dehydrogenation of methane for various transition metals, including nickel and copper. The total dissociation of methane on Cu and the other coinage metals was found to be very endothermic. Nickel was one of the most efficient catalysts for the methane dissociation described by sequential dehydrogenation of CH_4_:

4 $$ CH_{x,s \, } \to {\text{CH}}_{x - 1,s} + {\text{ H}}_{2} $$

In this work Cu–Ni bimetallic combinations with different Cu:Ni ratios were investigated with respect to their activity for methane activation and their tendency towards coking. The catalysts were thoroughly characterized regarding the Cu–Ni interaction by X-ray absorption and FTIR spectroscopy to confirm alloy formation. FTIR spectroscopy was utilized to characterize the surface composition and available adsorption sites by CO adsorption after oxidation and reduction in H_2_ as well as after methane exposure, in order to obtain insights into the effect of Cu addition to Ni catalysts. Cu addition to Ni/ZrO_2_ strongly reduced coking, while modification of the support surface by CeO_2_ addition did not have an effect in the present investigations.

## Materials and Methods

### Sample Preparation

Starting from Ni/ZrO_2_, materials of increasing complexity in composition were synthesized. First, Cu was added in different molar ratios. Additionally, the support surface was modified by CeO_2_. The Cu/Ni–CeO_2_/ZrO_2_ catalysts of different compositions as summarized in Table 1 were prepared by impregnation. One sample was obtained by another synthesis route, the combustion synthesis, to evaluate the influence of the synthesis procedure.

**Table 1:** Composition of Cu/Ni–CeO_2_/ZrO_2_ catalysts

Sample name	ZrO_2_ (wt%)	CeO_2_ (wt%)	Cu (wt%)	Ni (wt%)
Cu–Zr	95	–	5	–
31CuNi–Zr	95	–	3.75	1.25
11CuNi–Zr	95	–	2.5	2.5
13CuNi–Zr	95	–	1.25	3.75
Ni–Zr	95	–	–	5
Cu–CeZr	90	5	5	–
31CuNi–CeZr	90	5	3.75	1.25
11CuNi–CeZr	90	5	2.5	2.5
13CuNi–CeZr	90	5	1.25	3.75
Ni–CeZr	90	5	–	5

Commercial Zr(OH)_4_ (MEL chemicals XZO 880/01) was calcined with a heating rate of 2 K/min from room temperature to 973 K and kept at this temperature for 2 h. After calcination the zirconia support (Zr) was cooled down to room temperature.

For obtaining ceria/zirconia, Ce(NO_3_)_3_·6H_2_O was dissolved in water and ZrO_2_ was suspended in this solution to obtain a powder with 5.3 wt% CeO_2_. The suspension was dried over night at 373 K. Then the yellowish ceria/zirconia powder (CeZr) was heated to 723 K with a heating rate of 5 K/min and held there for 2 h. Addition of ceria by impregnation is expected to result in a modification of the oxide support surface, with the ceria mostly present on the surface.

Bimetallic CuNi samples were prepared by coimpregnation. Cu(NO_3_)_2_·3H_2_O and Ni(NO_3_)_2_·6H_2_O were mixed to obtain the following Cu:Ni molar ratios: 1:0, 3:1, 1:1, 1:3 and 0:1. The nitrates were dissolved in water and in each case 4.75 g ZrO_2_ or CeO_2_/ZrO_2_ powder was suspended in these solutions. Every solution contained as much metal nitrate to obtain 5 wt% metal in the final catalyst powder.

One catalyst sample was produced by a combustion synthesis route adapted from Ringuedé et al. [21]. The combustion catalyst had the same composition as sample 11CuNi–CeZr and will be designated CuNi–CeZr_C in the following. In this procedure, ZrO(NO_3_)_2_·6H_2_O, Ce(NO_3_)_3_·6H_2_O, Ni(NO_3_)_2_·6H_2_O and Cu(NO_3_)_2_·3H_2_O were mixed together and melted. Water was admixed to the molten mass and urea was added. Only half of the urea was necessary for the reaction while the rest was used as a fuel. After the addition of urea the mixture was placed into the preheated oven, where it was kept for 15 min at 873 K. After the impetuous reaction greenish agglomerates with a texture different from that of the other samples were obtained. Finally, fine powder was gained by hand-milling of the agglomerates.

### Characterization

X-ray diffraction (XRD) studies were carried out on an XPERT-PRO diffractometer with Cu K_α_ radiation operating at 40 kV and 40 mA with a 2θ scanning from 5° to 90° and a step size of 0.02°.

In situ X-ray absorption spectroscopy (XAS) measurements were carried out in fluorescence mode at the SuperXAS beamline [22] at the Swiss Light Source in Villigen, Switzerland using a Johann-type high-energy-resolution X-ray emission spectrometer and a single-photon-counting Pilatus 100 pixel detector. The spectrometer and experimental setup are described in detail in [23, 24]. The catalyst was placed between two plugs of quartz wool in a quartz plug flow reactor [25] which could be heated by an air blower heater. High energy resolution fluorescence detected X-ray absorption spectroscopy (HERFD-XAS) was measured at the maximum of the Cu K_α_ edge using a spherically bent Si(111) analyser crystal (R = 1,000 mm). The spectra of the X-ray absorption near edge structure (XANES) at the Ni K edge were acquired in total fluorescence yield mode using a PIPS diode. The catalyst was heated in a mixture of 5 % hydrogen in helium with a heating rate of 10 K/min from room temperature up to 660 K in case of Ni–Zr and up to 560 K for Cu–Zr and CuNi–Zr. Spectra were recorded at 300, 360, 430, 500, 560 and in case of Ni–Zr at 660 K. The sample was kept for about 20 min at each temperature while spectra were recorded. After cooling down to room temperature another spectrum at 300 K was recorded. X-ray absorption data were processed and analyzed using the IFEFFIT [26] software package.

The specific surface areas (BET) and porosity of calcined samples were calculated from N_2_ adsorption data acquired at liquid N_2_ temperature on a Micromeritics ASAP 2020 instrument. The powders were first outgassed at 573 K and <13 **×** 10^−3^ mbar for 60 min to ensure a clean surface prior to acquisition of the adsorption isotherm.

In order to determine the specific surface area of nickel measurements of hydrogen chemisorption were performed on a Micromeritics ASAP 2020C. Prior to analysis the samples were heated in an oxygen flow up to 773 K and kept at that temperature for 60 min. After cooling down in vacuum to 573 K the samples were heated in a hydrogen flow up to 673 K. After 30 min reduction the samples were evacuated for 30 min at 673 K. Chemisorption was performed at 308 K at hydrogen pressures between 75 and 775 mbar and repeated once in order to isolate the chemisorption isotherm. H_2_ was chosen as analysis gas because it adsorbs irreversibly on nickel but not on copper. Between the first and the repeated analysis the samples were evacuated at 308 K to remove reversibly adsorbed H_2_. The difference of the first isotherm data (reversible + irreversible adsorption) and the repeated isotherm data (only reversible adsorption) was utilized to calculate the quantity of irreversibly adsorbed hydrogen. For the determination of the metal dispersion hydrogen pressures of 150, 177, 212, 245 and 277 mbar were selected.

Surface sites and oxidation states of the metals were investigated by FTIR spectroscopy of chemisorbed CO. IR spectra were recorded in transmission using a Bruker Vertex 70 spectrometer with a mercury cadmium telluride (MCT) detector. Samples were pressed to small discs and placed in the IR cell. All infrared spectra were collected at a resolution of 4 cm^−1^ in the 4,000–900 cm^−1^ range by averaging 128 scans to achieve good signal to noise ratios. The results shown herein are difference spectra where the spectrum of the clean sample before adsorption was taken as a background.

The oxidation treatment was carried out in the IR cell by heating at 10 K min^−1^ from room temperature to 773 K under 100 mbar O_2_ pressure and holding that temperature for 1 h. Then, the IR cell was evacuated, and spectra were recorded before and after exposure to 5 mbar CO at 303 K. For measuring CO adsorption on reduced catalysts, the samples were heated in 50 mbar H_2_ and 900 mbar N_2_ at 10 K/min to 673 K after oxidation and kept at that temperature for 30 min. Then, the samples were outgassed at 673 K for 30 min (p ~10^−6^ mbar). Spectra before and after exposure to 5 mbar CO were recorded at 303 K.

IR spectra before and after exposure to 5 mbar CO were also recorded after heating a pre-oxidized sample in 5 mbar CH_4_ and 900 mbar N_2_ at 2 K min^−1^ to 773 K. The same procedure was applied to the pre-reduced samples.

## Results and Discussion

### Structural and Textural Properties

**Fig. 1:**
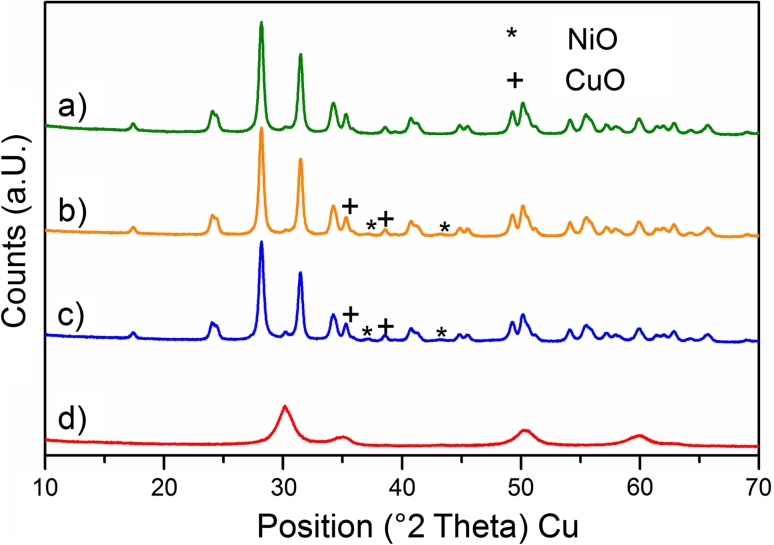
XRD patterns of *a* Zr, *b* 11CuNi–Zr, *c* 11CuNi–CeZr and *d* CuNi–CeZr_C after calcination. *a*–*c* show characteristics of monoclinic zirconia whereas *d* shows characteristics of a tetragonal structure

The crystalline structure was determined by XRD. The X-ray diffractograms of the samples Zr, 11CuNi–Zr, 11CuNi–CeZr and CuNi–CeZr_C after calcination are shown in Fig. 1. The pure ZrO_2_ support and the catalysts produced by impregnation show reflections assigned to monoclinic ZrO_2_, whereas the diffractogram of CuNi–CeZr_C is characteristic of tetragonal ZrO_2_. Apart from the different support phase the diffractograms also differ in reflection width. The much broader reflections of the combustion catalyst originate from a smaller support crystallite size, which is reasonable in light of the preparation procedure. Bulk CuO and NiO were not detected in the diffractograms, though. Both species seem to have too small crystallite size. Furthermore, bulk CuO has reflections very close to the characteristic reflections of monoclinic ZrO_2_ (monoclinic ZrO_2_: 35.4° and 38.7°; CuO: 35.6° and 38.8°).

In order to determine the oxidation state of the analysed copper and nickel containing samples, Ni K edge XANES and Cu K-edge HERFD-XAS spectra were recorded. NiO and Ni foil as well as CuO, Cu_2_O and Cu foil were analysed and served as reference spectra. The spectra obtained on the catalysts during reduction were fitted by a linear combination of these reference spectra to estimate the fraction of atoms in the respective oxidation states.

**Fig. 2:**
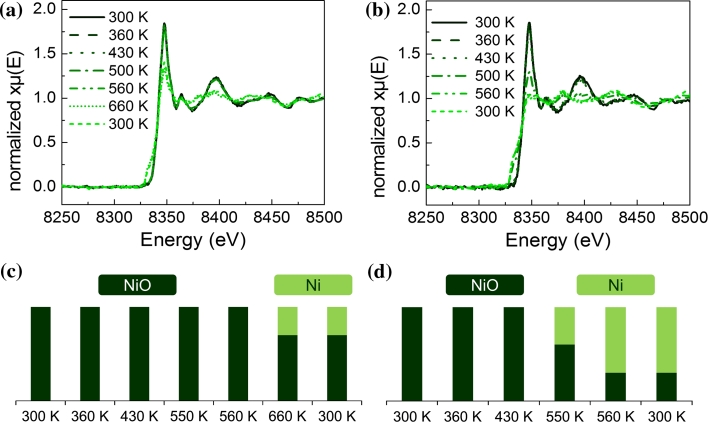
XANES spectra at the Ni K edge of **a** Ni–Zr and **b** 11CuNi–Zr during reduction in 5 % hydrogen in helium and amount of reduced versus oxidized nickel calculated by linear combination of reference spectra for **c** Ni–Zr and **d** 11CuNi–Zr

Figure 2 shows XANES spectra in H_2_/He at different temperatures during reduction. Both the Ni in Ni–Zr and in 11CuNi–Zr is completely oxidized until 430 K. On Ni–Zr nickel reduction starts at 660 K and the linear combination analysis indicates about 30 % of nickel atoms in the reduced (metallic) state. On the bimetallic catalyst 11CuNi–Zr reduction already sets in at 500 K. The amount of reduced nickel increases from 40 % at 500 K to 70 % at 560 K. This indicates that an alloy between Cu and Ni has formed under these conditions and Cu causes a decrease of the reduction temperature of Ni.

**Fig. 3:**
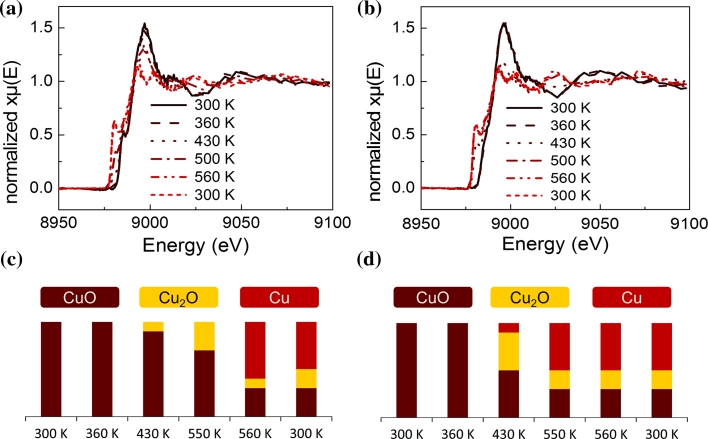
HERFD-XAS spectra at the Cu K edge of **a** Cu–Zr and **b** 11CuNi–Zr during reduction in 5 % hydrogen in helium and amount of reduced versus oxidized copper species calculated by linear combination of reference spectra for **c** Cu–Zr and **d** 11CuNi–Zr

Considering Cu–Zr and CuNi–Zr, a similar effect of lowering the reduction temperature of Cu was found as shown in Fig. 3. Up to 360 K copper was completely in the +II oxidation state in both mono- and bimetallic samples. After reduction at 430 K about 10 % of the monometallic copper was reduced to Cu(I)oxide while on the bimetallic sample 10 % were already reduced to Cu^0^, 40 % were in +I and 50 % in +II oxidation state. After reduction at 550 K the amount of Cu(I)oxide increased to 30 % in the monometallic sample, whereas 50 % Cu^0^, 20 % Cu_2_O and 30 % CuO were present in the bimetallic sample. Finally, after reduction at 660 K both catalysts were reduced to the same extent with 50 % Cu^0^.

The surface area analysis by N_2_ physisorption was carried out for the samples Zr, 11CuNi–Zr, 11CuNi–CeZr and CuNi–CeZr_C. The results of the calculations of the specific surface areas using the method of Brunauer, Emmet, and Teller (BET) and of the pore size and pore volume using the method of Barret, Joyner and Halenda (BJH) [27] are shown in Table 2.

**Table 2:** BET surface area und BJH average pore size and volume of Zr, 11CuNi**–**Zr, 11CuNi**–**CeZr and CuNi**–**CeZr_C

Sample name	BET surface area (m²/g)	BJH average pore size (nm)	BJH average pore volume (cm³/g)
Zr	36.6	21	0.18
11CuNi–Zr	35.1	20	0.17
11CuNi–CeZr	34.2	17	0.14
CuNi–CeZr_C	32.4	11	0.05

The highest surface area was found for ZrO_2_. By adding metal oxides by impregnation the specific surface area decreased by around 4–7 % likely due to blocking of some pores. The surface area of the combustion catalyst was significantly lower. The mesopores volume, calculated by the BJH method, was higher for the pure support and the impregnation catalysts than for the combustion catalyst.

### Adsorption Properties

#### H_2_ Chemisorption

The accessible nickel surface area was determined by selective chemisorption of H_2_. The active nickel surface areas per gram sample and per gram nickel are shown in Table 3. As expected, on the catalyst Cu–Zr no hydrogen adsorbs irreversibly, since this sample does not contain nickel. When comparing the accessible nickel surface area per gram nickel there is an enormous decrease for samples containing copper compared to Ni–Zr, while there is hardly any difference between the samples containing different Cu:Ni ratios (1:3, 1:1 and 3:1). The decrease of the nickel surface area per gram sample is not that enormous but also significant. These results can be explained by enrichment of Cu at the surface of the bimetallic materials in hydrogen after a reduction treatment at 673 K.

**Table 3:** Nickel surface area determined by Hydrogen Chemisorption

Sample name	wt% Ni	Nickel surface area (m^2^/g of sample)	Nickel surface area (m^2^/g of nickel)
Ni–Zr	5.00	1.570	31.40
13CuNi–Zr	3.75	0.140	3.74
11CuNi–Zr	2.50	0.067	2.67
31CuNi–Zr	1.25	0.036	2.84
Cu–Zr	0	–	–
CuNi–CeZr_C	2.50	–	–

When applying H_2_ chemisorption measurements for characterizing bimetallic catalysts, one has to be aware of complications that can arise from spillover processes. Evidence had been presented by Goodman and co-workers [28–30] that hydrogen can spillover from Ru or Re to Cu on Cu/Ru(0001) and Cu/Re(0001) surfaces. Hydrogen spilling over from Ni to Cu would imply more adsorbed hydrogen than nickel surface atoms. However, we detected a strongly reduced amount of hydrogen that can be adsorbed. Even though the results of the H_2_ chemisorption measurements are probably not fully accurate due to potential spillover effects, semi-quantitative information can be obtained and the Cu enrichment at the surface is clearly observed. To confirm this conclusion additional information was obtained from utilizing spectroscopic methods (see Sect. 3.2.3).On the catalyst CuNi–CeZr_C prepared by combustion synthesis no irreversible hydrogen adsorption is observed, in contrast to the impregnation catalysts. That implies that no reduced nickel is present on the surface of this sample after reduction in hydrogen at 673 K. By this synthesis procedure NiO, CuO, ZrO_2_ and CeO_2_ were formed simultaneously in one step. This may mean that NiO and CuO are incorporated into the support oxide lattice, and hydrogen is not able to reduce NiO at 673 K to Ni. XRD patterns in Fig. 1 did not show any reflections for NiO, but a different ZrO_2_ modification for CuNi–CeZr_C, which supports this interpretation. For Pt/CeO_2_ adsorption and reaction properties of materials prepared by combustion synthesis were found to be different than those of impregnated samples [31, 32].

#### Infrared Spectroscopy of CO Adsorption on Ni**–**Zr Catalysts

**Fig. 4:**
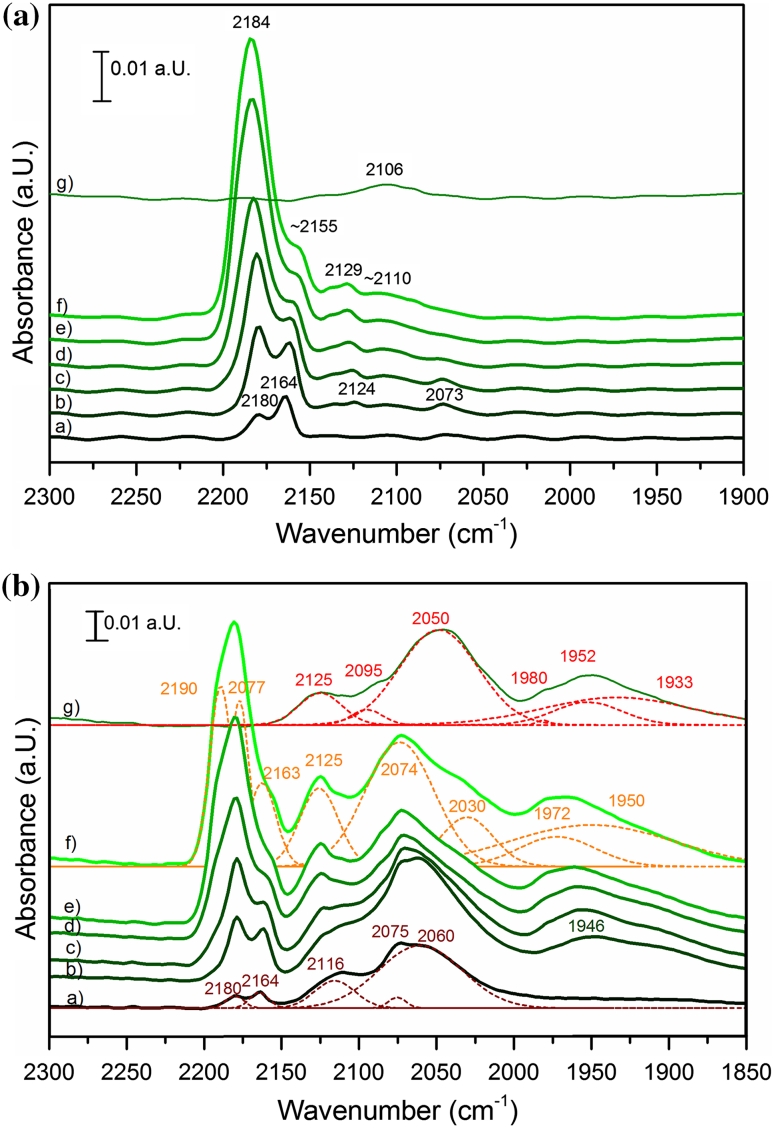
**A** IR Spectra of CO on Ni–Zr oxidized in 100 mbar O_2_ at 773 K: in *a* 0.005, *b* 0.2, *c* 0.5, *d* 1.0, *e* 3.0, *f* 5.0 mbar CO pressure and *g* after evacuation. **B** IR Spectra of CO on Ni–Zr reduced in 5 mbar H_2_ at 673 K: in *a* 0.005, *b* 0.2, *c* 0.5, *d* 1.0, *e* 3.0, *f* 5.0 mbar CO pressure and *g* after evacuation

Infrared spectra of room temperature CO adsorption on oxidized Ni–Zr are shown in Fig. 4a. According to literature data [8, 9, 33–35] adsorption of CO on ZrO_2_ results in a band at about 2,190 cm^−1^. Ni in different oxidation states can be distinguished by the vibration frequencies of adsorbed CO. Bands ascribed to Ni^n+^–CO surface complexes cover a broad spectral range including, besides linear monocarbonyls, also bridged, di- and tricarbonly species. Ni^2+^–CO produces a weak band at around 2,150–2,180 cm^−1^ [8, 34, 36–38]. CO forms stable monocarbonyls with Ni^+^ (2,160–2,100 cm^−1^) [39–42], which may be converted into dicarbonyls (ν_s_(CO) at 2,131–2,145 and ν_as_(CO) at 2,100–2,083 cm^−1^) [39, 41–43] with increasing CO pressure. The stretching vibrations of Ni^+^(CO)_3_ are expected at about 2,156, 2,124 and 2,109 cm^−1^ [39]. Linear carbonyls of metallic nickel appear at 2,050–2,094 cm^−1^ [36, 42–48] and bridged carbonyls are visible below 1,960 cm^−1^ [36, 44, 48].

Figure 4a displays spectra obtained in different CO background pressures to deduce additional information from changes with increasing CO coverage. At 0.05 mbar CO pressure two bands are visible: The Zr^4+^–CO interaction at 2,180 cm^−1^ and the Ni^2+^–CO interaction at 2,164 cm^−1^. At 0.2 mbar besides these two bands, absorption peaks at 2,124 and at 2,073 cm^−1^ can be observed. Those bands are attributed to the symmetric and antisymmetric CO stretching vibrations of Ni^+^(CO)_2_. With increasing pressure the low frequency band shifts to higher wavenumber and disappears. The three bands at about 2,155, 2,129 and 2,110 cm^−1^ are attributed to Ni^+^(CO)_3_. The band of the Ni^2+^–CO interaction is covered by the 2,155 cm^−1^-band of Ni^+^(CO)_3_ and the Zr^4+^–CO interaction, which increased strongly with increasing CO pressure and shifted to 2,184 cm^−1^. After evacuation only one band at 2,106 cm^−1^ remains. This band is attributed to Ni^+^–CO, which is known to be stable against evacuation [39, 41–43]. In summary, various CO on Ni^2+^ and Ni^+^ adsorption complexes can be observed in the spectra.

Infrared spectra of CO adsorption at various pressures obtained after reduction of Ni–Zr at 673 K are shown in Fig. 4b. Like on the oxidized sample, a Ni^2+^–CO peak at 2,164 cm^−1^ and a Zr^4+^–CO peak at 2,180 cm^−1^ are observed at 0.05 mbar CO pressure. With increasing CO pressure, the Zr^4+^–CO peak grows and covers the Ni^2+^–CO peak.

The band at 2,075 cm^−1^ appearing in 0.05 mbar CO is attributed to linearly adsorbed CO on Ni^0^, while the band at 1,946 cm^−1^, which starts to show up at 0.2 mbar, is attributed to bridge-bonded CO on Ni^0^. This band blue-shifts with increasing CO pressure. The vibration at 2,116 cm^−1^ shifting to 2,125 cm^−1^ with increasing pressure is attributed to Ni^+^–CO. The unusual oxidation state Ni^+^ can be stabilized by the ligand CO [41]. After evacuation Ni^+^–CO as well as linear and bridged CO on Ni^0^ remain on the surface. Overall, CO adsorption indicates that both reduced and oxidized Ni species are present on the surface. In agreement with the XAS measurements presented in Fig. 2, NiO reduction is not complete at 673 K.

#### Infrared Spectroscopy of CO Adsorption on Bimetallic Catalysts: Influence of Composition

**Fig. 5:**
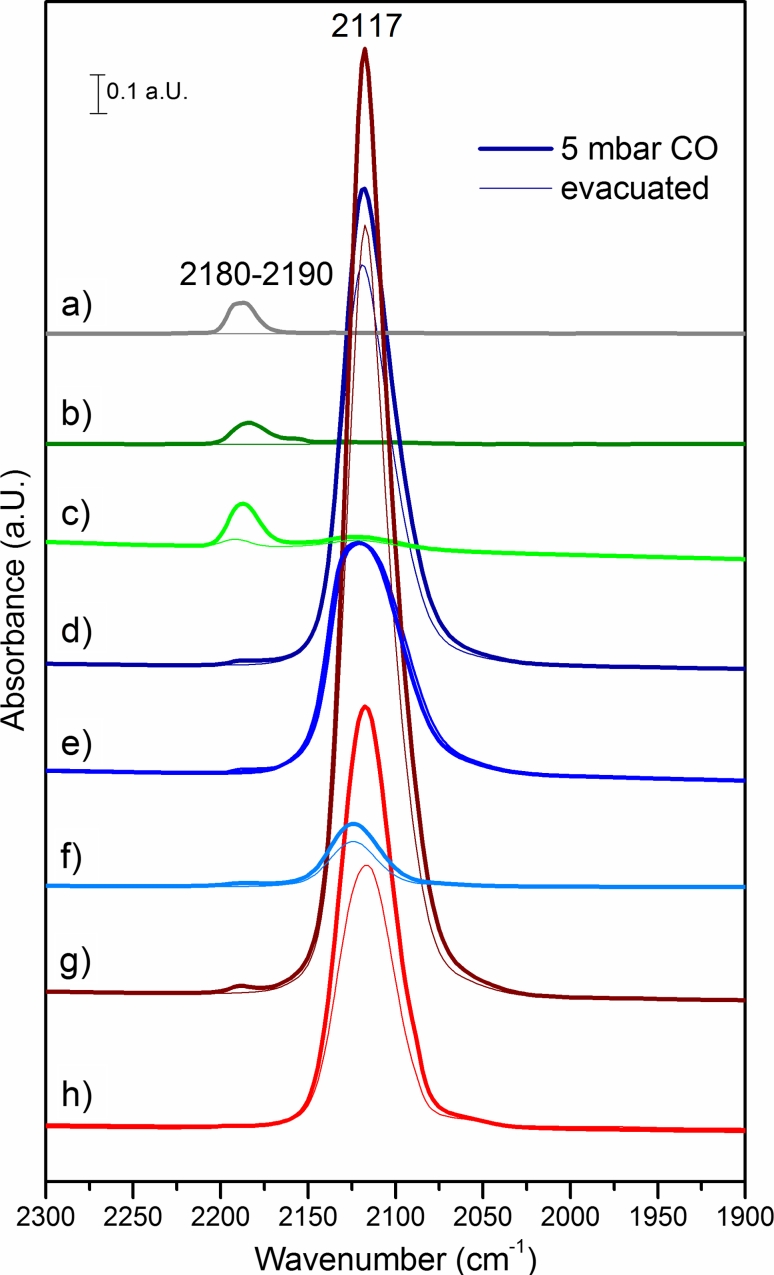
IR spectra of 5 mbar CO adsorbed on *a* Zr, *b* Ni–Zr, *c* Ni–CeZr, *d* 11CuNi–Zr, *e* 11CuNi–CeZr, *f* CuNi–CeZr_C, *g* Cu–Zr and *h* Cu–CeZr after oxidation in 100 mbar O_2_ at 773 K for 1 h

Infrared spectra of room temperature CO adsorption on the various oxidized samples are compared in Fig. 5. The spectra obtained in 5 mbar CO atmosphere are displayed in bold lines, fine lines characterize the CO which remains adsorbed after evacuation of gas phase CO at room temperature. Starting from Ni–Zr the effect of Cu addition and of the modification of the support by adding CeO_2_ on the surface adsorption sites was investigated.

First, CO was adsorbed on the pure zirconia and the ceria-zirconia. In good agreement with literature data [8, 9, 33–35] adsorption of CO on pure ZrO_2_ gives rise to a band at about 2,190 cm^−1^, which characterizes Zr(IV)–CO adsorption complexes [8, 33, 34]. The addition of CeO_2_ should cause a red-shift of this peak due to the lower vibrational frequency of CO on CeO_2_ [33], but as in this case the amount of ceria is rather low, the peak shift is negligible (spectrum not shown for brevity).

At about 2,150–2,180 cm^−1^ the absorption band of the weak Ni(II)–CO [8, 34, 36–38] interaction should appear, but as the weak bands of Ni(II)–CO and Zr(IV)–CO are very close to each other they cannot be distinguished at a CO pressure of 5 mbar. This was shown in more detail in Sect. 3.2.2 where different CO-species on oxidized Ni–Zr were detected by performing CO adsorption at lower CO pressures and coverages.

The only strongly interacting CO species observed on oxidized samples is the Cu^+^–CO interaction at around 2,115–2,120 cm^−1^. The Cu^+^–CO band was reported at around 2,120–2,143 cm^−1^ [49–51]. This interaction is much stronger than Cu^0^–CO appearing below 2,110 cm^−1^ [49–52] and Cu^2+^–CO at around 2,170–2,175 cm^−1^ [51]. On the bimetallic catalysts an absorption band of strongly adsorbed CO appears at around 2,120 cm^−1^ like on the copper monometallic catalyst, but the peaks are broader, and a slight shift to higher wavenumber can be observed. This may indicate an interaction of copper with neighbouring nickel atoms. The broader peaks suggest that there is a distribution of Cu(I) sites with slightly different electronic properties or chemical environment on the surface.

On the CuNi–CeZr_C sample the CO absorption band is even broader than on the 11CuNi–CeZr sample, and there is also a larger shift to higher wavenumbers with the CO vibrational band appearing at 2,124 cm^−1^.

Overall, CO adsorbed on Cu^+^ is the predominant species observed in the IR spectra on all Cu-containing samples.

**Fig. 6:**
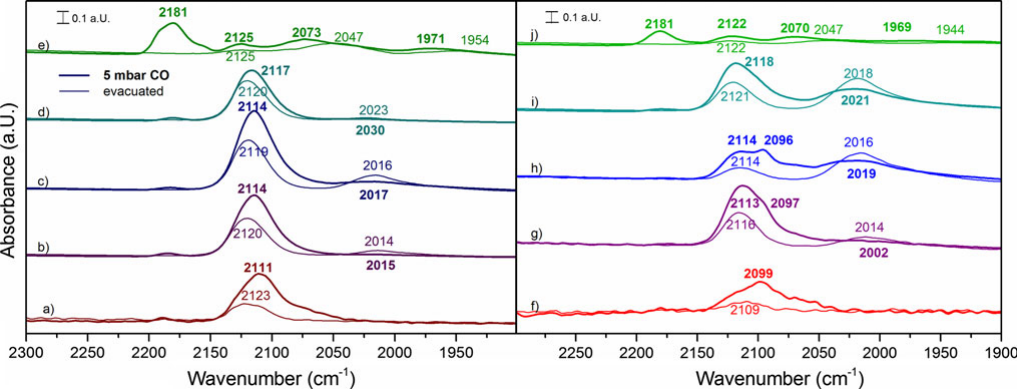
Infrared spectra of reduced catalysts in 5 mbar CO pressure and after evacuation of CO: *a* Cu–Zr, *b* 31CuNi–Zr, *c* 11CuNi–Zr, *d* 13CuNi–Zr and *e* Ni–Zr; *f* Cu–CeZr, *g* 31CuNi–CeZr, *h* 11CuNi–CeZr, *i* 13CuNi–CeZr and *j* Ni–CeZr

Infrared spectra of room temperature CO adsorption on reduced samples are shown in Fig. 6. Compared to the spectra of CO on oxidized samples, new bands have appeared. However, bands of CO on oxidized Cu and Ni species, which already existed on the oxidized samples, are still present indicating incomplete reduction.

On the monometallic nickel samples Ni–Zr and Ni–CeZr several new bands appeared, which are attributed to carbonyls on Ni^0^ and Ni^+^, as described in Sect. 3.2.2. On the copper containing samples the much weaker CO interaction with reduced Cu^0^ is observed in addition to the Cu^+^–CO band. CO on reduced Cu–Zr shows beside the Zr^4+^–CO interaction only one peak at around 2,111 cm^−1^ consisting of both Cu^0^–CO and Cu^+^–CO contributions, which shifts to 2,123 cm^−1^ after evacuation with mostly the more stable Cu^+^–CO remaining adsorbed at the surface.

**Scheme 1:**
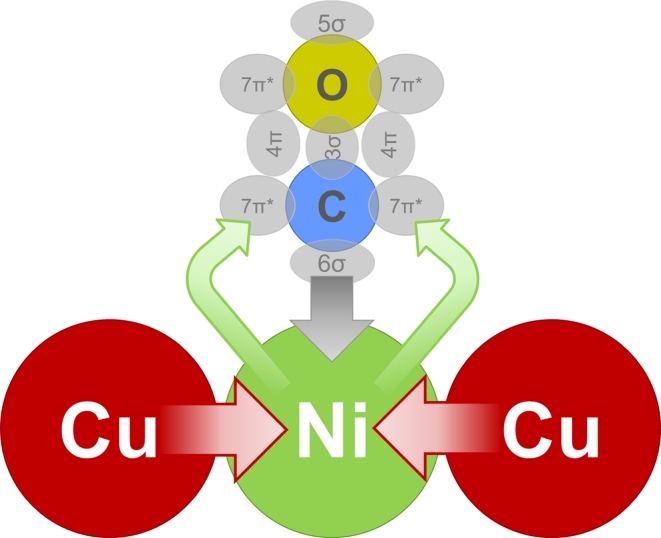
Schematic illustration of CO adsorption on CuNi alloys. Donation of d-electrons from copper to nickel leads to reinforcement of the π-backbonding from nickel to antibonding 7π* orbitals of CO. This leads to a weaker C–O bond and a red-shift of the CO stretch vibration band in infrared spectra

Upon Cu addition to Ni catalysts the Ni^0^–CO peak, which appears at about 2,070 cm^−1^ in 5 mbar CO and at about 2,050 cm^−1^ upon evacuation of gas phase CO on the Ni–Zr, red-shifts by approximately 50–70 cm^−1^ on the bimetallic samples to 2,012–2,023 cm^−1^ (Fig. 6). According to Blyholder’s scheme [48], the CO stretching vibration is predicted to shift from pure Ni as compared to Ni–Cu, because metallic Cu is expected to donate electrons to Ni. This should increase back-donation into the π* orbitals of CO with consequently lowering of the CO stretching frequency. Simultaneously, the electron donation reinforces the bond between CO and Ni. This is illustrated in Scheme 1. Compared to pure Ni, the CO bands on CuNi alloy were reported to shift to lower frequencies by about 40 cm^−1^ [48, 53]. Dalmon et al. [44] observed band of the irreversibly adsorbed Ni–CO at 2,058 cm^−1^ on monometallic nickel, at 2,028 cm^−1^ on a 36.6 % and at 2,005 cm^−1^ on a 72 % copper containing alloy, in good agreement with the present work. The fact that there is no shift of the Cu^+^–CO vibration with composition indicates that Ni is interacting with Cu^0^, but not with Cu^+^.

CO adsorption spectra were again recorded in different CO pressures to obtain information on coverage dependence of CO bands/species, as on the monometallic Ni–Zr catalyst. The band positions of the CO stretching vibrations on the bimetallic catalysts are summarized in Table 4. Beside the blue-shift of CO on nickel by 50–70 cm^−1^ with increasing Ni content, as described above, a blue-shift of 7–26 cm^−1^ with increasing CO coverage is observed. The vibration frequency of CO on the alloyed Ni^0^ blue-shifts by 7 cm^−1^ on copper-rich and up to 34 cm^−1^ on nickel-rich alloys with increasing CO coverage. After evacuation the CO vibration frequency is observed in between 2012 and 2,023 cm^−1^ on all samples. The stretching frequency of CO on Cu^+^ stays at about 2,110–2,120 cm^−1^ on all zirconia based catalysts and seems only little pressure dependent.

**Table 4:** CO stretch vibration frequencies on bimetallic CuNi**–**ZrO_2_ samples at different CO pressures

Sample	CO pressure (mbar)	CO on Cu/Cu^+^ (cm^−1^)	CO on Ni (cm^−1^)	Sample	CO pressure (mbar)	CO on Cu/Cu^+^ (cm^−1^)	CO on Ni (cm^−1^)
31CuNi–Zr	0.05	2,110	–	31CuNi––CeZr	0.05	2,104	1,995
	0.2	2,110	2,001		0.2	2,104/2,096	2,000
	0.5	2,111	2,011		0.5	2,107/2,097	2,005
	1	2,113	2,016		1	2,110/2,097	2,001
	3	2,113	2,018		3	2,110/2,097	2,000
	5	2,114	2,015		5	2,113/2,097	2,002
	Evacuated to <10^−5^	2,120	2,014		Evacuated to <10^−5^	2,116	2,012
11CuNi–Zr	0.05	2,110	–	11CuNi–CeZr	0.05	2,104	1,989
	0.2	2,110	2,000		0.2	2,114/2,095/2,076	2,000
	0.5	2,110	2,007		0.5	2,114/2,095/2,074	2,008
	1	2,113	2,014		1	2,112/2,096	2,013
	3	2,114	2,018		3	2,113/2,096	2,014
	5	2,114	2,017		5	2,114/2,096	2,019
	Evacuated to <10^−5^	2,119	2,016		Evacuated to <10^−5^	2,115	2,016
13CuNi–Zr	0.05	2,111	–	13CuNi–CeZr	0.05	2,107	1,997
	0.2	2,111	2,004		0.2	2,111/2,096/2,071	2,013
	0.5	2,112	2,013		0.5	2,115/2,096/2,073	2,015
	1	2,113	2,021		1	2,117/2,098	2,015
	3	2,116	2,025		3	2,117/2,098	2,017
	5	2,117	2,030		5	2,119	2,021
	Evacuated to <10^−5^	2,120	2,023		Evacuated to <10^−5^	2,121	2,019

In general, hardly any differences are observed between the zirconia and the ceria-zirconia supported catalysts. When comparing the zirconia supported catalysts with the ceria-zirconia based materials, which are shown on the left and right panel of Fig. 6, respectively, the shape of the peaks attributed to Cu^+^–CO and Cu^0^–CO is different. The CO stretching vibration on Cu–CeZr appears at a lower wavenumber than on Cu–Zr. After evacuation the band on Cu–CeZr blue-shifts from 2,099 to 2,109 cm^−1^ and decreases almost completely, while on Cu–Zr the band shifts from 2,111 to 2,123 cm^−1^ upon evacuation. This indicates that copper particles on reduced Cu–CeZr are interacting with CeO_2_, which seems to result in different electronic properties. Chen et al. [50] reported two peaks at 2,106 cm^−1^ and 2,078 cm^−1^ on Cu–Ce–O, which were assigned to the CO adsorption on two different linear adsorption sites. By doping with ZrO_2_ only one peak at 2,113 cm^−1^ was reported [50]. A band at 2,097 cm^−1^ was found by Manzoli et al. [52] and attributed to CO on small three dimensional metallic copper particles.

On the bimetallic samples 31CuNi–CeZr and 11CuNiCe–Zr two CO bands below and above 2,100 cm^−1^ are clearly visible, while the spectra on Ni–CeZr and 13CuNi–CeZr show hardly any difference to the zirconia supported samples. At low CO coverage a shoulder appeared at approximately 2,071–2,076 cm^−1^ on the nickel-rich ceria/zirconia supported samples 13CuNi–CeZr and 11CuNi–CeZr. This feature is in between the band at 2,078 cm^−1^ on Ce–Cu–O reported by Chen et al. [50] and the peak at 2,067 cm^−1^ on ceria and ceria/zirconia supported catalysts reported by Manzoli. In both cases this band was only found in presence of ceria. According to the interpretation of Manzoli et al. [52] this band is attributed to very small copper clusters, in close contact to reduced ceria. It seems as if ceria may give rise to copper sites with different electronic properties or even cause reduction of copper to a greater extent than on zirconia supported samples. Nickel properties were not changed by the addition of ceria.

Regarding the influence of the preparation procedure, the infrared spectra of CO on the reduced combustion catalyst CuNi–CeZr_C, which are not shown here, appear to be identical as after oxidation. This is in agreement with H_2_-chemisorption, where no indication of reduction of Ni under these conditions was found.

Clearly, in all samples the CO spectra are dominated by the signals of CO adsorbed on Cu. This implies a Cu enriched surface on the bimetallic samples, which is in agreement with chemisorption measurements.

#### Temperature Stability of CO Adsorption Complexes

**Fig. 7:**
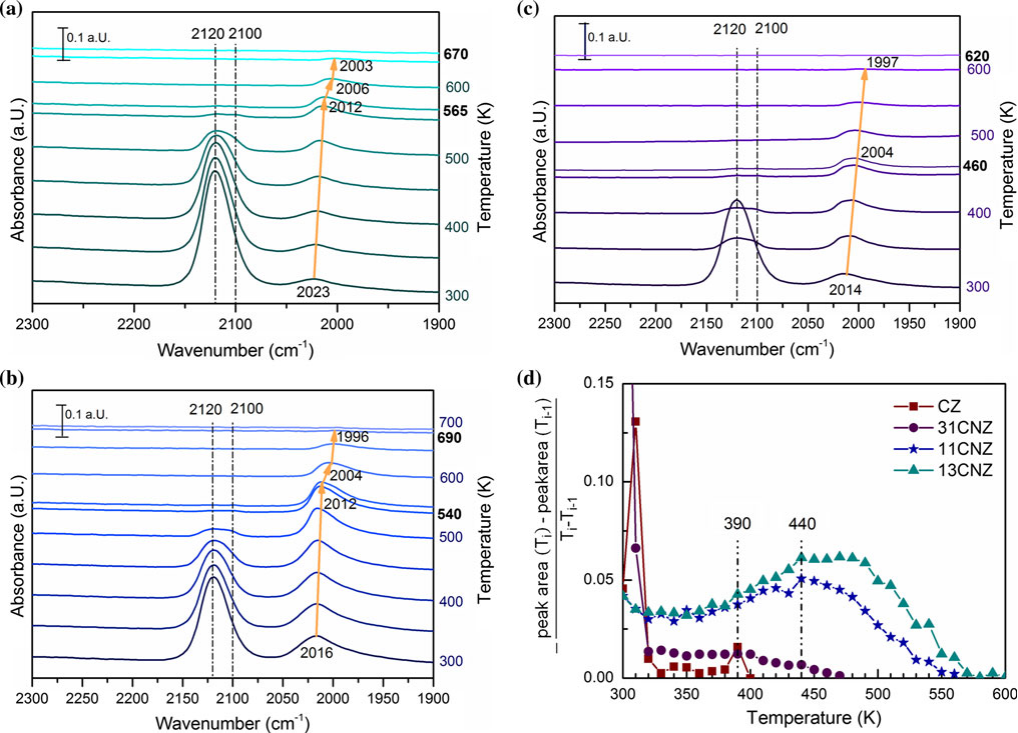
**a** Temperature programmed desorption of CO followed by FTIR spectroscopy on 13CuNi–Zr, **b** on 11CuNi–Zr and **c** on 31CuNi–Zr. **d** Area change of the integrated Cu–CO peak area of Cu–Zr, 31CuNi–Zr, 11CuNi–Zr and 13CuNi–Zr with temperature

Temperature stability of the adsorbed CO species on different samples was followed by recording IR spectra during heating with a rate of 10 K/min in vacuum. CO desorbed from Ni–Zr at about 450 K and from Cu–Zr at 410 K. On all bimetallic samples the desorption temperatures were higher, as shown in Fig. 7a–c.

With decreasing coverage the peak of CO adsorbed on copper species does not shift in frequency, but a second CO peak at about 2,100 cm^−1^ from Cu^0^–CO, which might be obscured by the Cu^+^–CO peak at about 2,120 cm^−1^ at room temperature due to the higher coverage, becomes visible with increasing temperature. On 13CuNi–Zr these two CO species are still present on the surface at 550 K, but disappear at about 565 K. On 11CuNi–Zr those peaks disappear between 540 and 550 K, and on 31CuNi–Zr they already vanish at about 450–460 K. Thus, with increasing copper content the CO desorption temperature is more similar to the monometallic copper sample. The usually unstable Cu–CO interaction seems to be reinforced by nickel.

To visualize the different CO desorption properties of alloyed and monometallic copper, difference quotients of the integrated peak areas of the copper peak between 2,160 and 2,080 cm^−1^ are shown in Fig. 7d. Most of the CO desorbs from Cu–Zr and the copper-rich sample 31CuNi–Zr slightly above room temperature. Only a small amount of CO remains on the surface of Cu–Zr before desorbing at around 390 K. On the samples 11CuNi–Zr and 13CuNi–Zr most of the CO desorbs at 440 K and above, while on 31CuNi–Zr a small shoulder at 440 K can be observed. Beside the Cu–CO interaction of low temperature stability, which already vanishes after rising the temperature slightly above room temperature, a second Cu–CO species is observed starting to desorb at 440 K. This species might be attributed to CO on copper surrounded by nickel. As copper tends to donate d-electrons to nickel strengthening the interaction between CO and nickel [48], the electron density in copper is reduced, which normally has its d-band completely filled. Less electrons in the d-band may reinforce the σ-bonding between CO and the metal and thus lead to a higher stability of this complex.

In parallel with the disappearance of the CO peak on copper, the CO peak on nickel shows a sudden shift to a lower wavenumber on 11CuNi–Zr and 13CuNi–Zr. This seems to be a coverage effect, as much CO desorbs from the catalyst at this stage. The Ni–CO peak completely disappears at about 670 K on 13CuNi–Zr and at about 700 K on 11CuNi–Zr. On 31CuNi–Zr the position of the Ni–CO band does not seem to be affected by the complete disappearance of the Cu–CO band, as only a small amount of CO is present and thus desorbs from 31CuNiZr at elevated temperatures. Furthermore, Ni–CO desorbs already around 620 K from this sample.

The total band shift of about 20 cm^−1^ from room temperature to desorption temperature is also observed on Ni–Zr and is attributed to a coverage effect, probably accompanied by a temperature effect on the CO vibration frequency.

To sum up, the adsorption strength of CO on copper increases with decreasing copper content. The Ni–CO band shows the highest stability on the 11CuNi–Zr sample. On 31CuNi–Zr it is more stable than on Ni–Zr, but less stable than on the nickel-rich sample.

### Methane Decomposition

After a detailed characterization of the materials, which confirmed Cu–Ni alloy formation but suggesting a Cu enrichment at the surface for all bimetallic Cu:Ni compositions, the reaction with methane, for which these catalysts are typically used, was followed again by utilizing infrared spectroscopy.

#### Methane Decomposition Followed by CO Adsorption on Pre-oxidized Catalysts

Pre-oxidized samples were heated in 5 mbar methane and 900 mbar nitrogen up to 773 K and then cooled down to room temperature in the reaction mixture. Afterwards, the chamber was evacuated and CO adsorption was applied another time in order to compare the oxidation state of the catalysts before and after methane conversion and to detect available versus affected CO adsorption sites on the surface.

**Fig. 8:**
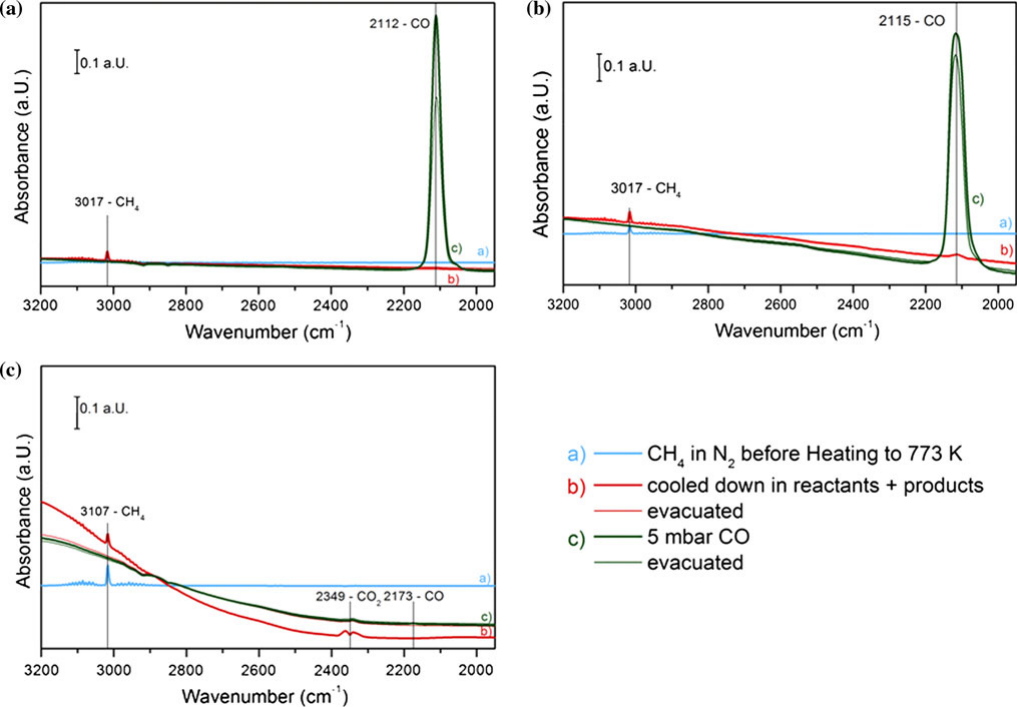
IR spectra recorded at room temperature before and after the methane heating ramp and subsequent CO adsorption on oxidized **a** Cu–CeZr, **b** 11CuNi–CeZr and **c** Ni–CeZr

Figure 8 shows infrared spectra before and after methane decomposition and during CO adsorption on pre-oxidized Cu–CeZr, 11CuNi–CeZr and Ni–CeZr. Generally, no difference was observed in zirconia supported samples, which are therefore not shown here.

On Ni–CeZr a strong change of the baseline is clearly visible in the FTIR spectra. This change in total absorption indicates a change of the sample’s colour most probably due to significant coke formation. A small amount of CO_2_ is formed during the reaction of methane giving rise to the characteristic IR band of CO_2_ in the gas phase. After methane exposure at 773 K CO adsorption is strongly decreased (Fig. 8c). Only about 6 % of the initially already small amount of CO adsorbing on a fresh oxidized sample were determined after this procedure. Since also the peak of the Zr^4+^–CO interaction decreases strongly, this could imply that not only nickel but also the support is partially covered by coke. In contrast, the IR spectra on Cu–CeZr before and after heating in methane to 773 K do not show differences as no IR active products are formed, and the baseline of the spectra is about the same. Compared to the fresh oxidized sample, 85 % CO can be adsorbed on this catalyst. The slight decrease may be attributed to copper sintering. On 11CuNi–CeZr little change in the total absorbance is observed and a small amount of CO is produced. Adsorption of CO is still possible after methane exposure, and compared to the fresh oxidized sample, 90 % CO adsorbs on the sample.

#### Methane Decomposition Followed by CO Adsorption on Pre-reduced Catalysts

**Fig. 9:**
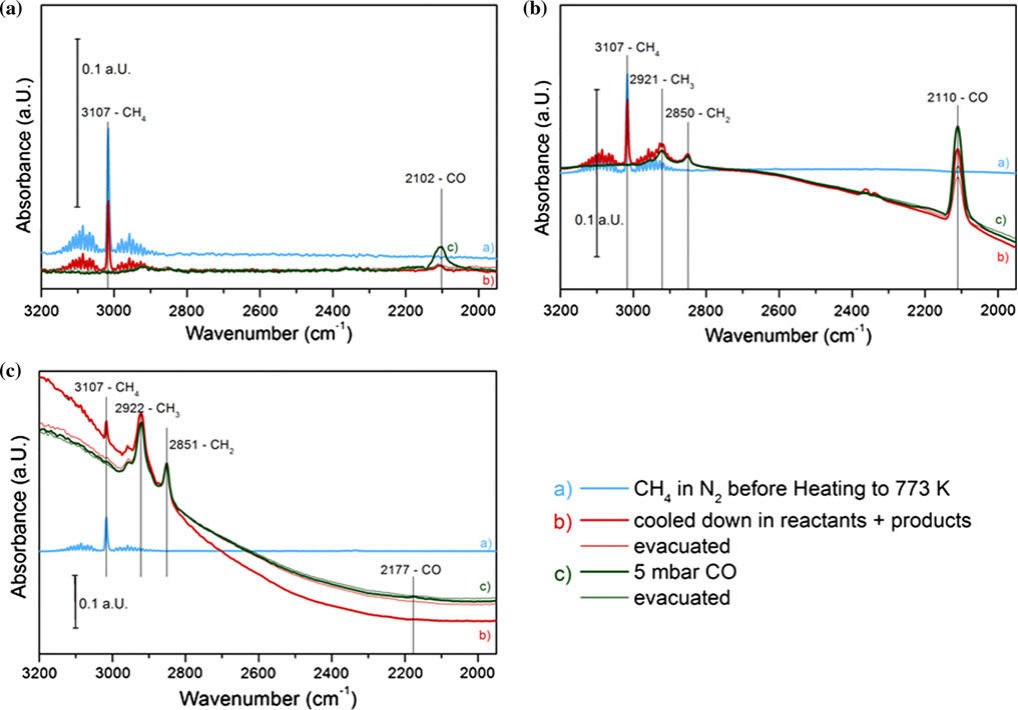
IR spectra recorded at room temperature before and after the methane heating ramp and subsequent CO adsorption on reduced **a** Cu–CeZr, **b** 11CuNi–CeZr and **c** Ni–CeZr

The same procedure as described in Sect. 3.3.1 was applied to the catalysts after reduction in H_2_/N_2_. IR spectra of pre-reduced Ni–CeZr, 11CuNi–CeZr and Cu–CeZr before and after methane decomposition followed by CO adsorption are shown in Fig. 9. The total absorption of the Ni–CeZr changed completely over the whole spectral range after methane decomposition, which again indicates considerable coke formation. Besides, a large amount of adsorbed CH_3_ and CH_2_ species formed. These species are intermediates of the partial dehydrogenation of methane. CO adsorption is practically impossible on the Ni sample. Only 1 % compared to the fresh reduced sample is adsorbed on Ni–CeZr after exposure to methane.

As on the pre-oxidized samples, Cu–CeZr appears to be much less reactive than Ni–CeZr and 11CuNi–CeZr. On Cu–CeZr a small amount of CO is produced out of methane. Compared to the sample directly after reduction in H_2_/N_2_ 40 % CO can adsorb after evacuation. Most probably, this can again be attributed to sintering of the now reduced copper taking place upon heating to 773 K. This suggests that on Cu–CeZr another mechanism of methane activation occurs than on nickel. On Cu–CeZr partial oxidation of methane occurs most probably with oxygen atoms from the support oxide while on nickel methane (partial) dehydrogenation is the main reaction.

On the bimetallic catalyst 11CuNi–CeZr CO, CO_2_ and adsorbed CH_3_ and CH_2_ are observed upon heating in methane (Fig. 9b). The amount of produced CO is much larger than on Cu–CeZr. Coking is apparently strongly reduced when regarding absorption over the whole spectral range recorded. When dosing CO after the methane exposure, in total about 40 % of the amount of CO that was adsorbed on a freshly reduced 11CuNi–CeZr sample was adsorbed on the used catalyst. From this it can be concluded that the bimetallic 11CuNi–CeZr catalyst is active for methane dehydrogenation but much less coke formation is detected than on Ni catalysts. On the bimetallic Cu–Ni catalyst both pathways are observed, but (partial) oxidation is favoured and dehydrogenation down to elemental carbon is strongly reduced.

This is in line with the observed enrichment of the surface in copper, breaking up larger nickel ensembles, which are supposed be responsible for coke formation, and/or changing the electronic properties of nickel resulting in reduced C–H bond breaking.

Gavrielatos et al. [18] and Triantafyllopoulos and Neophytides [19] reported reduced coke formation on Ni–YSZ modified with Au compared with unmodified Ni–YSZ. Generally, two parameters can be considered for the graphite development on nickel surfaces. Firstly, a critical ensemble on Ni is needed for graphite formation [54]. Au was found to be enriched on the surface breaking up larger Ni ensembles [18, 19]. Secondly, Besenbacher et al. [55] showed by DFT calculations and experimental measurements that small amounts of Au on the Ni surface increase the resistance toward carbon formation either by increasing the activation barrier of methane dehydrogenation reaction or by decreasing the binding energy of the resulting CH_x_ and C species on the Ni surface.

Liu et al. [56] recently performed DFT calculations for the methane dissociation on copper-rich NiCu(111) compared to Cu(111) and Ni(111). A decreased coke deposition on the copper rich alloy surface was predicted, because on the one hand the activation barrier of the rate determining step of methane dissociation is higher by 0.27 eV on copper-rich NiCu(111) than on Ni(111) but lower by 0.58 eV than on Cu(111), which means that a Cu-rich NiCu surface can suppress carbon deposition [56]. Another feature which was found to prevent the building up of a graphite layer was the decrease of C adsorption energy on copper-rich NiCu(111) compared to Ni(111) [56].

## Conclusions

In this work a detailed FTIR spectroscopic study of Cu and Ni bimetallic combinations supported on zirconia and ceria–zirconia is presented. Starting from Ni–Zr, the complexity in composition of the materials investigated was increased by adding Cu to the Ni particles and by modification of the support with ceria.

Adsorption of CO was applied to determine the oxidation state and available surface sites. Copper–nickel alloy formation was indicated by the red-shift of the CO stretching frequency on metallic nickel by about 30 cm^−1^ as well as by X-ray absorption measurements which revealed a lower reduction temperature of NiO and CuO in bimetallic alloyed samples as compared to monometallic catalysts. The extent of the red-shift of the Ni–CO band depends on the bulk composition. In addition, the composition affected the desorption temperature of CO on copper, resulting in significantly higher temperature stability on all the bimetallic samples.

Both hydrogen chemisorption and FTIR spectroscopy of CO adsorption clearly demonstrated that the surface of the bimetallic particles was strongly enriched in Cu with about the same composition for samples with different nominal copper:nickel bulk compositions.

Upon exposure to methane at 773 K, coke formation occurred over Ni–CeO_2_/ZrO_2_ resulting in a strong change in total absorption. In addition, CH_3_ and CH_2_ species were formed on the Ni catalyst, which proves (partial) dehydrogenation of methane. In contrast, on Cu–CeO_2_/ZrO_2_ CO production was observed with no further changes in the IR spectra. On the bimetallic 11CuNi–CeZr sample after reduction both CH_2_, CH_3_ and CO formation took place coming along with strongly reduced coke formation compared to Ni.

While Cu strongly influenced the reaction and adsorption properties of the Ni samples, ceria–zirconia materials exhibited very similar properties and surface chemistry as zirconia supported samples in the present study. The main difference was an additional IR band of CO adsorbed on metallic copper pointing to an interaction of part of the Cu with the ceria.
